# Genetic diversity of *Trichoderma atroviride* strains collected in Poland and identification of loci useful in detection of within-species diversity

**DOI:** 10.1007/s12223-015-0385-z

**Published:** 2015-03-20

**Authors:** Dominik Skoneczny, Michał Oskiera, Magdalena Szczech, Grzegorz Bartoszewski

**Affiliations:** 1 Department of Plant Genetics Breeding and Biotechnology, Warsaw University of Life Sciences (SGGW), Nowoursynowska 159, 02-776 Warszawa, Poland; 2 Microbiology Laboratory, Research Institute of Horticulture, Konstytucji 3 Maja 1/3, 96-100 Skierniewice, Poland; 3 Present Address: School of Agricultural and Wine Sciences, Charles Sturt University, Locked Bag 588, Wagga Wagga, NSW 2678 Australia

## Abstract

Molecular markers that enable monitoring of fungi in their natural environment or assist in the identification of specific strains would facilitate *Trichoderma* utilization, particularly as an agricultural biocontrol agent (BCA). In this study, sequence analysis of internal transcribed spacer regions 1 and 2 (ITS1 and ITS2) of the ribosomal RNA (rRNA) gene cluster, a fragment of the translation elongation factor 1-alpha (*tef1*) gene, and random amplified polymorphic DNA (RAPD) markers were applied to determine the genetic diversity of *Trichoderma atroviride* strains collected in Poland, and also in order to identify loci and PCR-based molecular markers useful in genetic variation assessment of that fungus. Although *tef1* and RAPD analysis showed limited genetic diversity among *T. atroviride* strains collected in Poland, it was possible to distinguish major groups that clustered most of the analyzed strains. Polymorphic RAPD amplicons were cloned and sequenced, yielding sequences representing 13 *T. atroviride* loci. Based on these sequences, a set of PCR-based markers specific to *T. atroviride* was developed and examined. Three cleaved amplified polymorphic sequence (CAPS) markers could assist in distinguishing *T. atroviride* strains. The genomic regions identified may be useful for further exploration and development of more precise markers suitable for *T. atroviride* identification and monitoring, especially in environmental samples.

## Introduction

*Trichoderma* is a fungal genus that includes more than 200 species that occur all over the world in different geographical regions and climatic zones (Atanasova et al. 2013; Hoyos-Carvajal et al. 2009; Kredics et al. 2014). They are remarkable for their rapid growth under various environmental conditions, as well as their ability to use different substrates as carbon sources (Kubicek et al. 2003; Atanasova and Druzhinina 2010); thus, they are recognized as efficient decomposers of woody and herbal materials and other organic matter (Atanasova et al. 2013; Schuster and Schmoll 2010). *Trichoderma* interacts with other microorganisms and plants in diverse ways (Harman et al. 2004; Lorito et al. 2010; Druzhinina et al. 2011; Woo et al. 2006). *Trichoderma* strains, including *Trichoderma atroviride* strains, that are promoting efficient plant growth and stress resistance are used as biological control agents (BCAs) in sustainable farming systems (Chet and Inbar 1994; Benítez et al. 2004; Stewart and Hill 2014).

Various molecular techniques have been used in *Trichoderma* research, in order to investigate genetic diversity within the genus. These include restriction fragment length polymorphism (RFLP) analysis (Dodd et al. 2004a), random amplified polymorphic DNA (RAPD) analysis (Hermosa et al. 2001), amplified fragment length polymorphism (AFLP) analysis (Buhariwalla et al. 2005), sequence-characterized amplified region (SCAR) analysis (Hermosa et al. 2001; Dodd et al. 2004b; Cordier et al. 2007), microsatellite markers (Naef et al. 2006), and sequence analysis (Kindermann et al. 1998; Dodd et al. 2003; Druzhinina et al. 2005; Błaszczyk et al. 2011; Atanasova et al. 2013). One of the simplest and cheapest techniques is RAPD, which is characterized by low repeatability and transferability between different laboratories. Nevertheless, it delivers solid data at minimal expense when used strictly in a single laboratory. Moreover, RAPD markers can be easily transformed into PCR-based SCAR markers, as demonstrated in several studies for *T. atroviride* (Hermosa et al. 2001; Cordier et al. 2007; Feng et al. 2011).

Application of molecular techniques in *Trichoderma* taxonomy solved inaccuracies related to phenotypical *T. atroviride* species identification, as well as demonstrated interspecies genetic variability. In order to reevaluate biocontrol strains reported as *Trichoderma harzianum* and *Trichoderma viride*, Hermosa et al. (2000) utilized sequences of internal transcribed spacer regions 1 and 2 (ITS1 and ITS2) of the ribosomal RNA (rRNA) gene cluster and analyzed the hybridization patterns of mitochondrial DNA*.* Authors identified four groups of *Trichoderma* including *T. atroviride*. Identification of mycoparasitic strains performed with assistance of ITS1, ITS2, mtSSUrDNA, and partial *chi18-5* gene sequences revealed that the strains belonged to *T. harzianum*, *T. atroviride*, and *Trichoderma asperellum* (Kullnig et al. 2001). Based on the sequences of ITS1, ITS2, and translation elongation factor 1-alpha (*tef1*) gene fragments, four major clades (A, B, C, and D) of *T. atroviride* were distinguished, and the fifth clade E was suggested (Dodd et al. 2003; Jaklitsch et al. 2006; Samuels et al. 2006; Mulaw et al. 2010; Gal-Hemed et al. 2011).

Application of *Trichoderma* BCA strains raised questions about fungal survival and spread in the environment (Dodd et al. 2004a, b). The molecular strategies were applied to develop markers useful for *Trichoderma* monitoring at different levels: genus-specific (Hagn et al. 2007; Devi et al. 2011; Chakraborty et al. 2011; Friedl and Druzhinina 2012), species-specific (Chen et al. 1999; Kredics et al. 2009; Miyazaki et al. 2009; Friedl and Druzhinina 2012), and strain-specific (Hermosa et al. 2001; Rubio et al. 2005; Cordier et al. 2007; Savazzini et al. 2008). Strain-specific molecular markers proposed for *Trichoderma* BCA strain monitoring included RAPD, RFLP (Zimand et al. 1994; Bowen et al. 1996; Abbasi et al. 1999), and SCAR markers (Hermosa et al. 2001; Dodd et al. 2004b; Rubio et al. 2005; Cordier et al. 2007; Savazzini et al. 2008; Longa et al. 2009; Feng et al. 2011; Naeimi et al. 2011). Due to the complex taxonomy of *Trichoderma*, development of strain-specific markers is a long and laborious process (Druzhinina and Kubicek 2005). The most beneficial is development of species-specific markers, which can also be used to simplify and assist in taxonomic identification, based on sequence differences at the interspecies level, useful for elaboration of such markers (Chen et al. 1999; Friedl and Druzhinina 2012; Kredics et al. 2009; Miyazaki et al. 2009).

The aim of this study was to utilize sequences of ITS1 and ITS2 region, a fragment of the *tef1* gene, and RAPD markers to analyze the genetic diversity of *T. atroviride* strains collected in Poland, develop PCR-based markers, and identify genomic regions to enable genetic diversity studies and further development of reliable methods of *T. atroviride* identification. In this study, RAPD markers were converted into PCR-based markers suitable for detection of *T. atroviride*. Additionally, application of CAPS markers developed in this study is expected to improve the accuracy of *T. atroviride* classification.

## Materials and methods

### Fungal material and strain identification

All *Trichoderma* strains used in this study were obtained from a stock culture collection maintained by the Microbiology Laboratory at the Research Institute of Horticulture in Skierniewice, Poland. Stock cultures were deep-frozen in glycerol and stored at −80 °C. Forty strains of *T. atroviride* were used in this study; these included 38 strains of *T. atroviride* collected at different locations in Poland, and the two reference strains CBS 693.94 (Dodd et al. 2003) and IMI206040 (Kubicek et al. 2011) (Table 1). Strain identification was based on the internal transcribed spacer regions 1 and 2 (ITS1 and ITS2) of the rRNA gene cluster sequences and *Trich*OKey v. 2.0 (Druzhinina et al. 2005) and sequences of the fragment of the gene encoding translation elongation factor 1-alpha (*tef1*) and *Tricho*BLAST v. 1.0 analysis (Kopchinskiy et al. 2005). All sequences were obtained by PCR and direct amplicon sequencing from both directions. ITS1 and ITS2 regions were PCR-amplified with ITS6 and ITS4 primers (Cooke and Duncan 1997; White et al. 1990). For the *tef*1 sequencing, primers EF1-728 F and TEFLLErev were used (Carbone and Kohn 1999; Jaklitsch et al. 2005). ITS and *tef1* sequences of the strains are available in the NCBI GenBank (Accession numbers are provided in Table 1). The following strains were used for the PCR-based marker species specificity evaluation: *Trichoderma aggressivum* f. *aggressivum* CBS 100528, *T. aggressivum* f. *europaeum* CBS 100526, *T. asperellum* TRS705, *Trichoderma citrinoviride* TRS119, *Trichoderma cerinum* 38.24.06.2, *Trichoderma crassum* TRS113, *Trichoderma gamsii* TRS123, *Trichoderma hamatum* TRS127, *T. harzianum* sensu lato II subclade CBS 466.94, *T. harzianum* s. l. III subclade TRS60, *T. harzianum* s. l. X subclade CBS 115901, *T. harzianum* s. l. Lixii subclade TRS66, *T. harzianum* sensu stricto TRS71, *Trichoderma longibrachiatum* TRS708, *Trichoderma pleuroticola* TRS120, *Trichoderma spirale* TRS111, *Trichoderma tomentosum* TRS82, *Trichoderma virens* Gv29-8, *Trichoderma viride* TRS575, *Trichoderma viridescens* TRS35, and *Trichoderma velutinum* 29.24.06.1. For all above mentioned strains, species/subclade affiliation was confirmed by ITS and *tef1* sequencing as described above for *T. atroviride*. Mycelia taken from stock cultures were placed on potato dextrose agar (PDA) plates (Sigma-Aldrich, St. Louis, MO, USA) and cultured in the dark at 25 °C for 4–7 days. Mycelia grown on PDA were then used to inoculate 250-mL flasks containing 50 mL potato dextrose broth (Sigma-Aldrich), and cultures were incubated in the dark on a rotary shaker at 200 rpm for 7 days at room temperature (21 ± 2 °C).

**Table 1:** Source and origin of *T. atroviride* strains used in this study

Strain	GenBank accession numer of sequences		Source	Location	Origin
	ITS	*tef1*			
TRS31	KJ786740	KJ786821	Soil	Balcerów	Central Poland
TRS15	KJ786724	KJ786805	Lumberyard	Głuchów	
TRS36	KJ786729	KJ786810	Soil	Kątne	
TRS28	KJ786721	KJ786802	Production hall	Kolonia Bolimowska	
TRS5	KJ786741	KJ786822	Compost	Łódź	
TRS2	KJ786749	KJ786830	Compost	Łódź	
TRS1	KJ786739	KJ786820	Mushroom growing hall	Maków	
TRS3	KJ786743	KJ786824	Mushroom growing hall	Maków	
TRS9	KJ786738	KJ786819	Mushroom growing hall	Maków	
TRS18	KJ786757	KJ786839	Compost	Maków	
TRS32	KJ786744	KJ786825	Production hall	Maków	
TRS45	KJ786718	KJ786799	Compost	Maków	
TRS20^a^	KJ786730	KJ786811	Production hall	Sierakowice Lewe	
TRS30	KJ786720	KJ786801	Soil	Skierniewice	
TRS14^a^	KJ786725	KJ786806	Compost	Skierniewice	
35.24.06.4^a^	KJ786742	KJ786823	Field soil	Skierniewice	
TRS68	KJ786745	KJ786826	Mushroom growing hall	Skierniewice	
TRS17	KJ786734	KJ786815	Mushroom growing hall	Skierniewice	
TRS21	KJ786727	KJ786808	Soil	Topołowo	
TRS22	KJ786726	KJ786807	Soil	Wiskitki	
TRS24	KJ786722	KJ786803	Production hall	Iganie Nowe	Eastern Poland
TRS25^a^	KJ786731	KJ786812	Production hall	Iganie Nowe	
TRS38	KJ786733	KJ786814	Soil	Myślibórz	Northern Poland
TRS39	KJ786728	KJ786809	Soil	Myślibórz	
TRS42	KJ786752	KJ786833	Soil	Radzanów	
TRS7^a,b^	KJ786747	KJ786828	Production hall	Bieruń	Southern Poland
TRS40^a^	KJ786755	KJ786836	Compost	Rzeczyce	
TRS26	KJ786751	KJ786832	Soil	Lipniak	Western Poland
TRS6	KJ786750	KJ786831	Compost	Sława	
TRS10	KJ786754	KJ786835	Not known	Turek	
TRS43^a^	KJ786748	KJ786829	Forest soil	Wielkopolski Park Narodowy	
TRS11	KJ786735	KJ786816	Not known	Wrocław	
TRS12^a^	KJ786737	KJ786818	Not known	Wrocław	
TRS13	KJ786736	KJ786817	Not known	Wrocław	
TRS16	KJ786753	KJ786834	Not known	Not known	Poland
TRS19	KJ786732	KJ786813	Soil	Not known	
TRS34	KJ786719	KJ786800	Not known	Not known	
TRS44	KJ786756	KJ786837	Not known	Not known	
CBS 693.94 (IMI 359825)	AF359263	KJ786838	Mushroom compost	*T. atroviride* reference strain^**c**^	Northern Ireland
IMI206040	AF278795.1	ABDG00000000.2	Biocontrol strain	*T. atroviride* reference strain^d^	USA

^a^Strains used in RAPD primer screening part of study

^b^Not included in main genetic diversity study

^c^Dodd et al. (2003)

^d^Kubicek et al. (2011)

### DNA extraction

Total fungal DNA was isolated using the DNeasy Plant Mini Kit (Qiagen, Hilden, Germany), according to the manufacturer’s instructions with slight modifications. Fungal cells of tested *Trichoderma* strains were harvested by centrifugation of liquid cultures and dried on filter paper at room temperature (21 ± 2 °C), prior to grinding in liquid nitrogen with quartz sand (Sigma-Aldrich). Proteinase K and RNase A were added to the lysis buffer. The purity and quantity of isolated DNA were evaluated by agarose gel electrophoresis followed by ethidium bromide staining, and by determination of A_260_/A_280_ using a NanoDrop 8000 spectrophotometer (Thermo Scientific, Waltham, MA, USA). Finally, the DNA concentration was adjusted to 1 ng/μL by diluting the samples with 10 mmol/L Tris-HCl, pH 8.0.

### RAPD analysis

A set of 520 decamer primers (primer kits OPA to OPZ from Operon Technology, Alameda, CA, USA) was used for initial RAPD screening of eight strains of *T. atroviride*: 35.24.06.4, TRS7, TRS12, TRS14, TRS20, TRS25, TRS40, and TRS43. Then, 68 primers that had given the best results were further tested on 39 strains to assess the genetic diversity of *T. atroviride* strains collected in Poland. RAPD-PCR was performed as described by Olczak-Woltman et al. (2009). Reactions contained 1× Green PCR Buffer containing 2 mmol/L MgCl_2_, 0.2 mmol/L each dNTP, 1 μg bovine serum albumin, 0.5 U *Taq* polymerase (Fermentas, Vilnius, Lithuania), 1 μmol/L arbitrary primer, and 2 ng DNA template. PCR was carried out under conditions of initial incubation at 95 °C for 1 min, followed by 10 cycles of 95 °C for 5 s, 37 °C for 30 s, and 72 °C for 30 s; 35 cycles of 95 °C for 5 s, 37 °C for 30 s, and 72 °C for 60 s; and a final extension at 72 °C for 7 min. RAPD-PCR was carried out in a PTC-200 Thermal Cycler (Bio-Rad, Hercules, CA, USA). Amplified DNA fragments were separated by electrophoresis with 2 % agarose–0.5× Tris-borate-EDTA (TBE) gels containing ethidium bromide and visualized under UV light. Gel images were edited in CorelDRAW version 12 (Corel Corporation, Ottawa, Ontario, Canada). The binary matrices were constructed based on evaluation of clear RAPD amplicons. Amplicons were scored for presence (1) or absence (0) for each strain. Cluster analysis of the binary data was performed using the NTSYS-pc v.2.1 program (Exeter Software, Setauket, NY, USA). Similarity matrices were generated using Jaccard’s coefficients, and an unweighted pair-group method using arithmetic averages (UPGMA) was chosen to generate the dendrogram from RAPD similarity matrices. To determine how faithfully the dendrogram preserves pairwise distances between original unmolded data, the cophenetic correlation coefficient parameter (*r*) was calculated.

### Cloning and sequencing

Polymorphic RAPD amplicons were excised from the gel, and DNA was purified using the QIAquick Gel Extraction Kit (Qiagen) according to the manufacturer’s protocol. Next, purified amplicons were reamplified under the conditions described for RAPD-PCR, resolved using a 1 % agarose–0.5× TBE gel, excised from the gel, and purified. Efficiently reamplified RAPD amplicons were cloned using a TOPO TA Cloning Kit (Invitrogen, Carlsbad, CA, USA). Recombinant plasmids were isolated, and two independent plasmids were used for sequencing of each amplicon (with the exception of Q01-420, for which only one clone was available) using universal M13 forward and reverse primers. The Sequencher 5.1 program (Gene Codes Corporation, Ann Arbor, MI, USA) was used to assemble single reads to obtain consensus sequences in FASTA format. The consensus sequences were deposited at the National Center for Biotechnology Information (NCBI), and GenBank accession numbers are summarized in Table 2 (RAPD-PCR amplicons) and Table 3 (CAPS amplicons).

**Table 2:** GenBank accession numbers and results of bioinformatics analysis of RAPD-PCR amplicon sequences

RAPD-PCR amplicon	NCBI GenBank Acc. No.	*T. atroviride* IMI 206040 v.2 unmasked genome BLAST hits (not filtered for low complexity)		*T. atroviride* IMI 206040 gene catalog (transcripts)		Protein NCBI BLAST hits (non-redundant database)		Pfam domains (database v. 27)
		scaffold	e-value	Similarity	e-value	Similarity	e-value	
A10-933	KF364331	contig_23	0.0	Triat2:317841	9.14E-8	NS	NA	Not found
B07-500	KF364332	contig_29	0.0	Triat2:302951	1.17E-132	Hypothetical protein	7e-42	Not found
E03-391	KF364333	contig_28	0.0	Not found	NA	NS	NA	Not found
E04-458	KF364334	contig_27	0.0	Not found	NA	NS	NA	Not found
H08-917	KF364335	contig_16	0.0	Triat2:254812	0.0	Tetratricopeptide (TPR) protein	8e-116	Tetratricopeptide repeat, PF13414 and PF07719
M06-768	KF364336	contig_22	0.0	Triat2:299234	9.13E-176	NS	NA	Not found
Q01-420	KF364337	contig_26	0.0	Not found	NA	NS	NA	Not found
Q01-885	KF364338	contig_24	0.0	Not found	NA	NS	NA	Not found
R08-1283	KF364339	contig_29	5.06e-53	Triat2:231658	5.05E-64	Putative ankyrin repeat-containing protein	1e-175	Ankyrin repeats (3 copies) PF12796
T07-429	KF364340	contig_27	0.0	Triat2:321796	6.01E-157	Putative ankyrin repeat-containing protein	7e-429	Not found
X16-1030	KF364341	contig_14	0.0	Triat2:172973	0.0	Hypothetical protein	3e-142	Magnesium transporter NIPA PF05653
X18-634	KF364342	contig_23	0.0	Triat2:299500	0.0	Hypothetical protein	3e-35	Not found
Z04-873	KF364343	contig_20	0.0	Triat2:291123	0.0	Hypothetical protein	6e-54	Not found

*NS* no significant similarity, *NA* not applicable

**Table 3:** Description of CAPS markers and reaction conditions

CAPS amplicon	Primer names and sequences^a^ (5′-3′)	Amplicon length (bp)^b^	Optimized PCR conditions (annealing temperature/number of cycles)	Restriction enzyme	GenBank Accession number/strain
Q01-4	Q01_4F-GCACACCAACTGCTGGAGCTT	1017	66 °C/27 cycles	*Bsl*I	KF576213/TRS15 KF576210/TRS22 KF576211/TRS40
	Q01_4R-CACGCTGACAATGACCGACAC				
X18-35	X18_3F-AGGCACAGTCCCCTGTTTAGT	358	65 °C/35 cycles	*Taq*I	KF576212/TRS36 KF576213/TRS42
	X18_5R-TGACGATCCTGGTAAGGTTTG				
Z04-2	Z04_2F-TTACCCAGTGCGGAATCCAAA	1450	66 °C/27 cycles	*Dra*I	KF576214/CBS 693.94 KF576215/TRS45
	Z04_2R-TATACGGCGCCTTCCACATTG				

*CAPS* cleaved amplified polymorphic sequence

^a^Sequences of PCR amplicons of representative strains were deposited at NCBI

^b^Amplicon length is based on genome sequence of *T. atroviride* reference strain IMI20604

### Bioinformatics analysis

For phylogenetic reconstruction, sequences of the fragment of *tef1* gene were assembled with ClustalX (Thompson et al. 1997) and manually edited in CLC Genomic Workbench 7.5 (CLCBio, Aarhus, Denmark), and final block alignment was prepared with a less stringent selection option using GBlocksServer (http://molevol.cmima.csic.es/castresana/Gblocks_server.html). The unconstrained GTR + I + G substitution model, estimated nucleotide frequencies, and substitution values were selected according to Samuels et al. (2006). Metropolis-coupled Markov chain Monte Carlo (MCMCMC) analysis was performed with two runs for one million generations with four chains, with the heating coefficient λ = 0.1 with MrBayes 3.2.2 × 64 (Ronquist et al. 2012).

RAPD amplicon sequences were compared using basic local alignment search tool (BLAST) (Altschul et al. 1997) with publicly available sequences deposited at both NCBI and the Genome Portal of the Joint Genome Institute (JGI, Grigoriev et al. 2012). At NCBI, blastn and blastx were used to explore the non-redundant protein sequence, nucleotide collection, and high-throughput genomic sequence databases. At JGI, sequences were aligned using blastn to the *T. atroviride* IMI206040 genome version 2 unmasked assemblies. Proteins containing amino acid sequences similar to those encoded by RAPD amplicons were collected and further analyzed using CLC Genomics Workbench 7.5 (CLCBio), and Pfam database search (Punta et al. 2012). RAPD sequences were scanned for sequence repeats using RepeatMasker (http://www.repeatmasker.org/) and mfold (Zuker 2003).

### Cleaved amplified polymorphic sequence marker development

The consensus RAPD sequences were mapped on the *T. atroviride* IMI 206040 v. 2 genome sequence deposited at JGI (http://genome.jgi-psf.org/Triat2/Triat2.home.html). Several sets of primers were designed to amplify RAPD regions or extended RAPD regions based on genome sequence. A standard PCR was performed using *Taq* polymerase (Fermentas) according to the manufacturer’s instructions. PCR was carried out under conditions of initial incubation at 94 °C for 3 min, followed by 35 cycles of 94 °C for 30 s, optimized annealing temperature or gradient temperature for 30 s, and 72 °C for 2 min, followed by a final extension at 72 °C for 10 min. The amplified products for selected *T. atroviride* strains were analyzed on agarose gels, purified, and sequenced. Derived sequences were compared and searched for polymorphic restriction sites using Sequencher 5.1 (Gene Codes). PCR amplification was performed using selected primer pairs. PCR products were digested with corresponding restriction enzyme (*Bsl*I, *Dra*I, and *Taq*I; all from Fermentas) according to the manufacturer’s instructions, and digestion products were separated on 1.5 % agarose–0.5× TBE gel. The respective primer pairs with optimized PCR conditions and corresponding restriction enzymes are shown in Table 3. To confirm specificity of the CAPS PCR to *T. atroviride* species, multiplex PCR was performed with DNA of 19 *Trichoderma* species mentioned above, CAPS primers, and additional primers 5.8S-R and LR6 (Vilgalys and Hester 1990). The pair of primers 5.8S-R and LR6 amplified a 1.45-kb fragment of the ITS region and served as a positive control for the PCR.

## Results and discussion

**Fig. 1:**
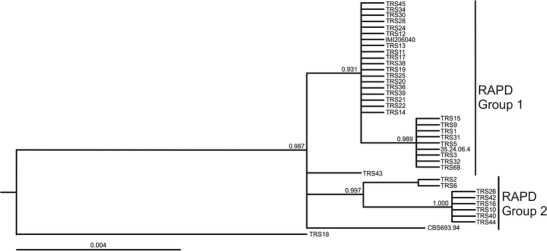
Bayesian identification phylogram of *T. atroviride* strains used in this study based on *tef1* gene sequence alignments. *Numbers* at nodes indicate the posterior probability coefficients obtained after one million generations. IMI206040 and CBS693.94 are reference strains of *T. atroviride*

Molecular identification of strains based on sequences of ITS1, ITS2, and the fragment of *tef1* gene confirmed that all of the strains used in this study belong to *T. atroviride*. Based on *tef1* sequences, all strains were classified to clade A described by Dodd et al. (2003), except for TRS18 that did not fit any clade. *Trich*OKEY 2 analysis revealed one new ITS1 sequence variant that possesses a single nucleotide change in comparison to the IMI206040 sequence. Six strains, TRS15, TRS24, TRS28, TRS30, TRS34, and TRS45, were characterized by the new ITS1 variant. Seven variants of *tef1* sequence which differed at least by a single nucleotide were found within the studied *T. atroviride* strains. Bayesian phylogeny analysis of *tef1* sequences allowed strain clustering (Fig. 1). TRS18 strain was found to be the most distant one. Four groups of strains were distinguished, with the two biggest groups differing within by a single nucleotide change only. Strains TRS43 and CBS 693.94 did not fall into any groups (Fig. 1).

**Fig. 2:**
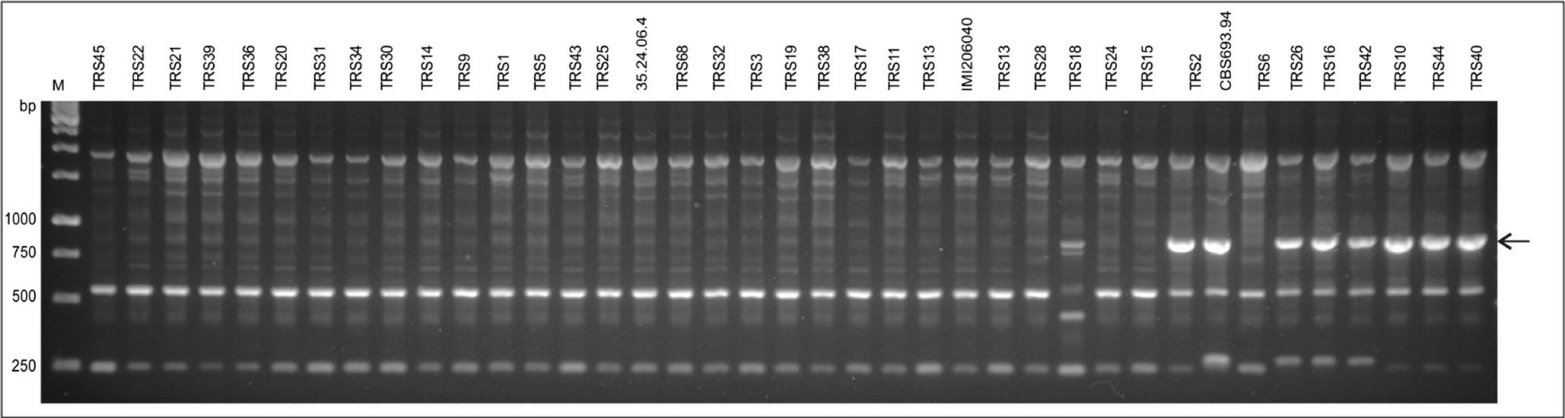
RAPD-PCR amplification profile generated with primer OPZ04 for *T. atroviride* strains used in this study. Polymorphic amplicon OPZ04-873 bp is shown by the *arrow*. IMI206040 and CBS 693.94 are reference strains of *T. atroviride. M*, 1-kb DNA size marker

In addition, RAPD markers were used for further evaluation of genetic diversity of *T. atroviride* strains. A total of 520 primers were tested on eight *T. atroviride* strains, including 35.24.06.4, TRS7, TRS12, TRS14, TRS20, TRS25, TRS40, and TRS43, in order to select RAPD primers useful for analysis. Amplicons were obtained for 482 primers, with an average of eight amplicons of different sizes identified for each primer. Based on electrophoretic evaluation of amplification pattern quality, 68 primers that produced polymorphic amplicons were chosen for the assessment of genetic diversity of the *T. atroviride* strains. RAPD PCR was performed with selected primers on 38 strains of *T. atroviride* collected at different locations in Poland (Table 1). As a control, *T. atroviride* reference strains CBS 693.94 (Dodd et al. 2003) and IMI 206040 (Kubicek et al. 2011) were used. High-quality amplification products were obtained for 55 of the 68 RAPD primers. Only two primers (OPK11 and OPX09) did not show any polymorphisms among tested strains. The greatest number of polymorphic amplicons distinguishing groups of strains was obtained with primer OPA10. Amplification profiles of three primers, OPI18, OPQ01, and OPZ04, showed that the *T. atroviride* strains, with the exception of strain TRS18, can be classified into two main groups. The amplification profile generated using OPZ04 is shown in Fig. 2.

**Fig. 3:**
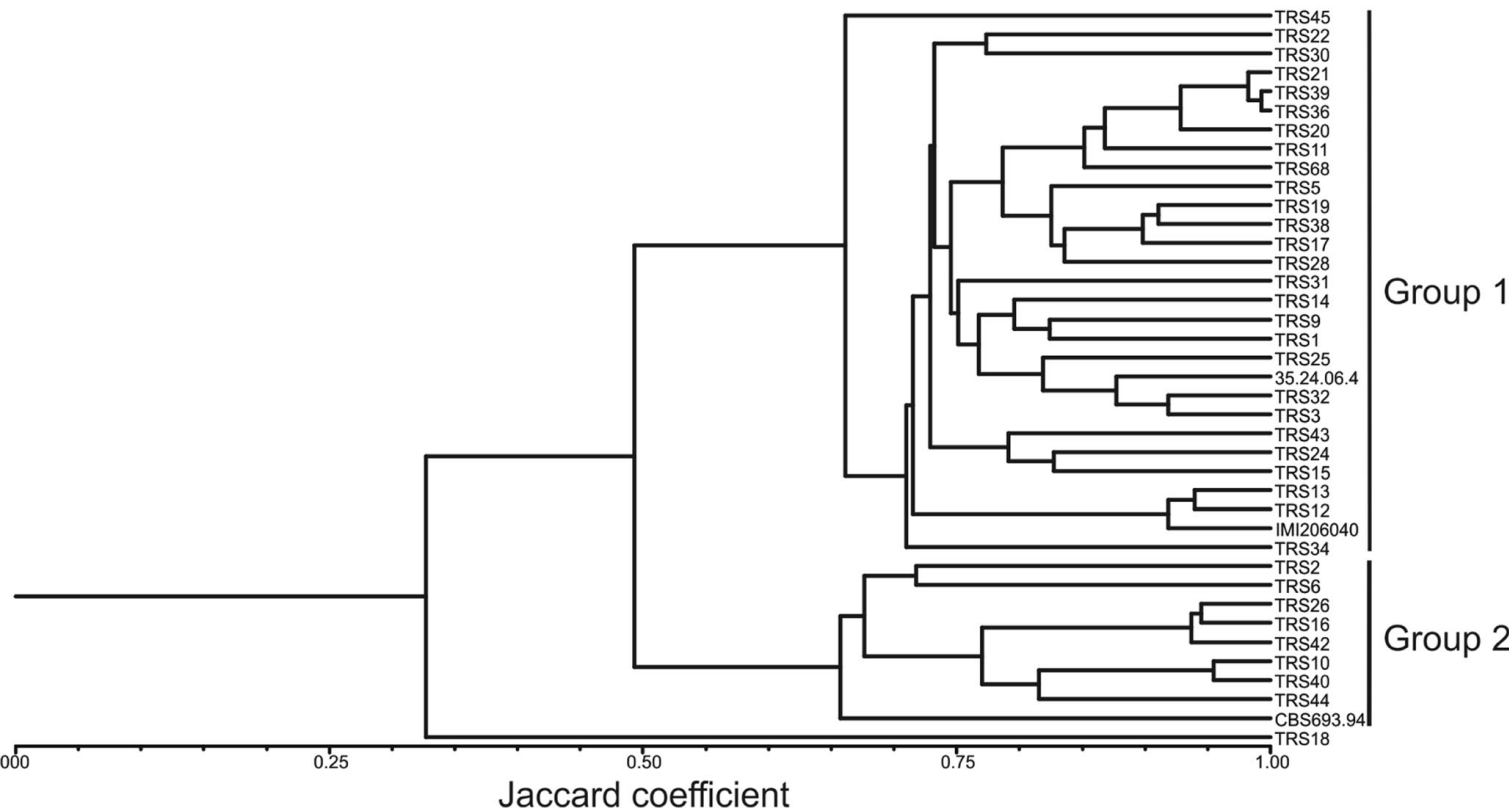
Dendrogram generated using Jaccard’s coefficient and the UPGMA clustering method. Binary matrices for *T. atroviride* strains were constructed based on evaluation of RAPD amplicons generated using 55 primers. Cluster analysis of the binary data was performed using NTSYS-pc 2.1 software. Similarity matrices were generated using Jaccard’s coefficient and an unweighted pair-group method using arithmetic averages (UPGMA) was used to generate the dendrogram

RAPD amplicons were scored for presence and absence, and 391 amplicons differentiating tested strains were used to construct a binary matrix, and then a dendrogram illustrating genetic diversity (Fig. 3). The cophenetic correlation coefficient (*r*) for the dendrogram was 0.979, indicating a good fit between the unmolded data and the dendrogram. Tested *T. atroviride* strains, of which all except TRS18 belonged to clade A, were classified into two main groups. The first group was represented by the reference strain IMI206040, as well as 28 of the tested strains, and corresponds to the two biggest groups of strains including TRS43, distinguished based on *tef1* sequence. The second group contained reference strain CBS 693.94 and the following eight tested strains: TRS2, TRS6, TRS10, TRS16, TRS26, TRS40, TRS42, and TRS44. This group corresponds to CBS 693.94 and remaining two small groups of strains distinguished based on *tef1*. The observed variation among strains within the two major groups was rather limited; however, some subgroups could be distinguished (Fig. 3). *T. atroviride* strain TRS18 was clearly distinct from both groups of strains, based on both *tef1* and RAPD analysis, and also did not fit any previously described clades. TRS18 *tef1* intron 4 sequence BLAST similarity search with non-redundant nucleotide sequence database at NCBI revealed 95 % identity with any sequence deposited in GenBank. This suggests that TRS18 strain is characterized by a unique and distinct genotype that could represent new clade of *T. atroviride*.

Twenty polymorphic RAPD markers were chosen for cloning, and 13 RAPD-PCR products were successfully cloned and sequenced (Table 2). Resulting sequences were aligned with the *T. atroviride* IMI206040 genome at the JGI portal. Twelve RAPD sequences were identical to corresponding *T. atroviride* IMI206040 sequences (e-value = 0). Sequence R08-1283 showed some variation, but the e-value was very low, indicating strong similarity to the *T. atroviride* genome (Table 2). Seven sequences showed strong similarity to hypothetical proteins of *T. atroviride* deposited in the NCBI database, and Pfam protein domains were clearly identified for three of these (Table 2). The H08-917 sequence showed a high degree of similarity to tetratricopeptide repeat (TPR)-containing proteins. TPR motifs are short amino acid repeats that occur in many proteins and not only are responsible for interactions between proteins but also facilitate protein complex formation and protein transport within cells. TPR-containing proteins play a role in protein assembly and therefore have a significant impact on the cell cycle and a plethora of cellular processes (D’Andrea and Regan 2003). Interestingly, the H08-917 sequence also possessed short and degenerate DNA repeats that were identified by RepeatMasker and mfold. The R08-1283 and T07-429 sequences were recognized as proteins with ankyrin (ANK) repeats, which play a role in protein-protein interactions (Rubtsov and Lopina 2000). Moreover, the X16-1030 sequence was similar to the NIPA membrane protein, which functions as a magnesium transporter (Goytain et al. 2007, 2008).

**Fig. 4:**
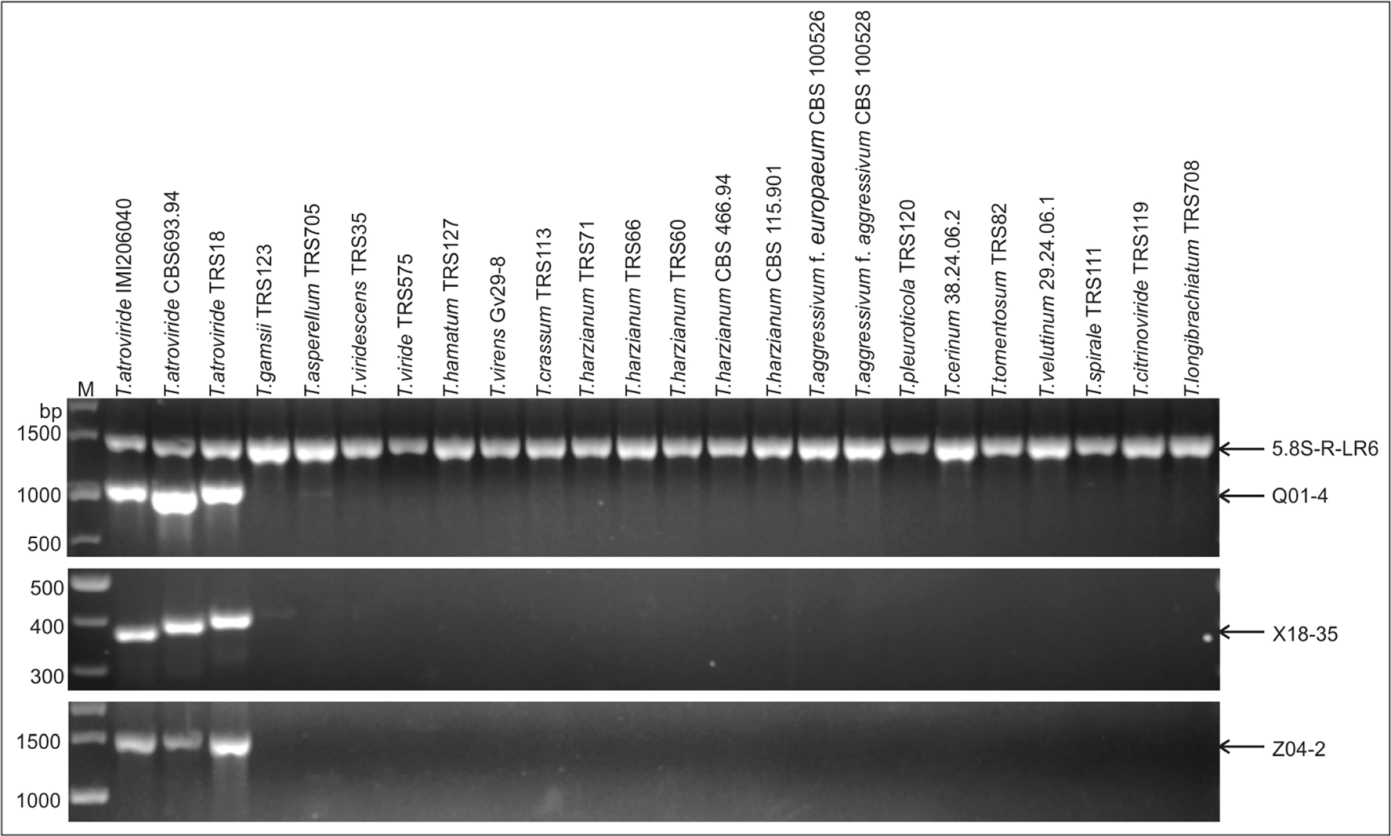
Amplification of Q01-4, X18-35, and Z04-2 fragments in different *Trichoderma* species. *M*, DNA size markers; amplicon 5.8S-R-LR6, PCR positive control

Using the identified RAPD marker sequences and the *T. atroviride* genome sequence, pairs of specific primers were designed, followed by optimization of PCR conditions and testing of the primers on a set of *Trichoderma* strains belonging to different species, including *T. atroviride* (Table 3). Unique PCR amplification products for three pairs of primers, Q01-4, X18-35, and Z04-2, were obtained for *T. atroviride* strains and not for 16 other *Trichoderma* species/clades (Fig. 4). In the case of X18-35, a very faint PCR amplicon was obtained for *T. gamsii*. These suggest that the amplicons Q01-4, X18-35, and Z04-2 are specific to *T. atroviride*. We propose that pairs of primers Q01-4, Z18-35, and Z04-2 could be used for *T. atroviride* detection.

**Fig. 5:**
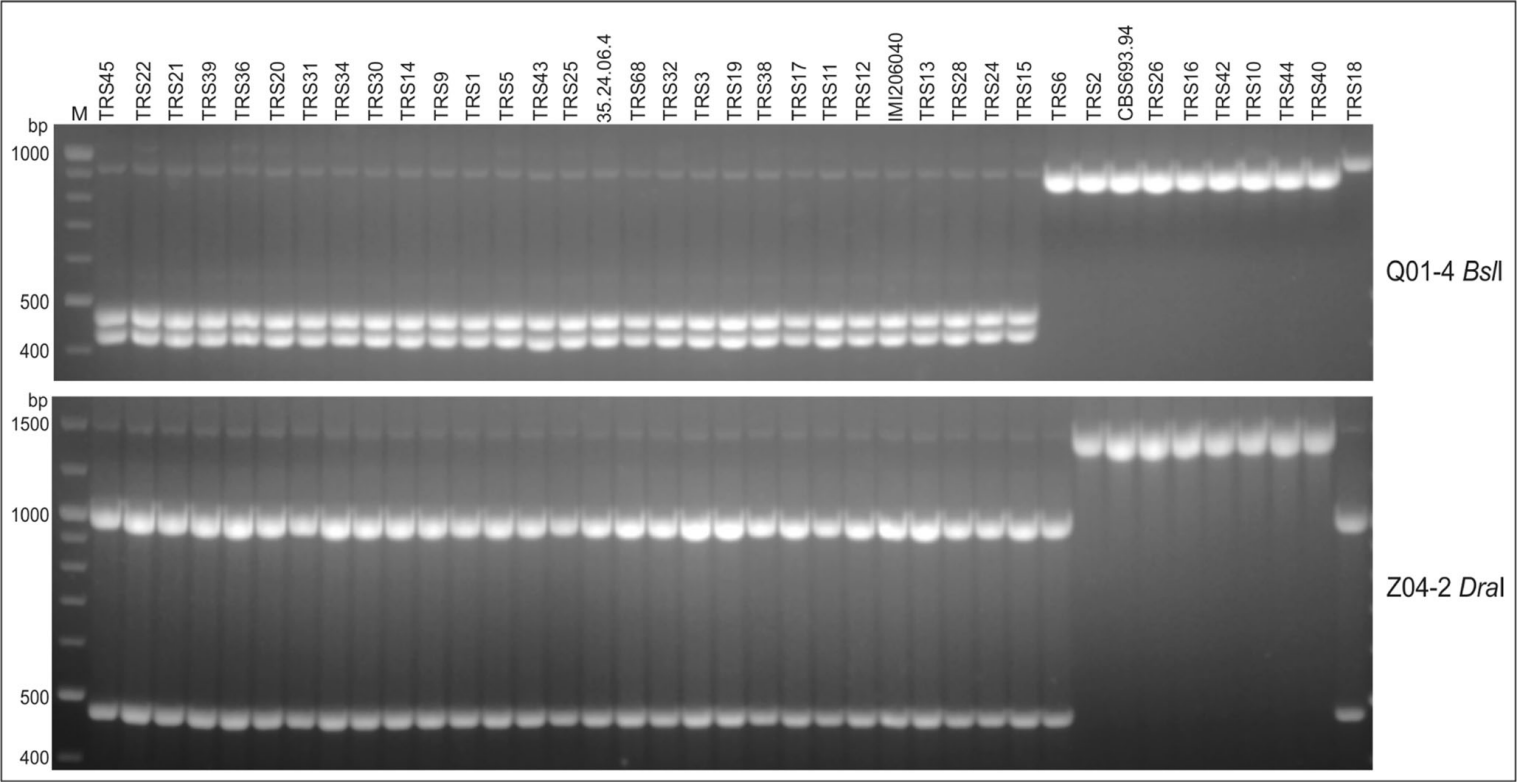
Q01-4 and Z04-2 CAPS marker profiles generated by PCR amplification with primers Q01_4F and Q01_4R and digestion of PCR products with *Bsl*I (*upper part*) and primers Z04_2F and Z04_2R and digestion of PCR products with *Dra*I. IMI206040 and CBS 693.94 are the reference strains of *T. atroviride. CAPS*, cleaved amplified polymorphic sequences. *M*, 100-bp Plus DNA ladder

For selected *T. atroviride* strains representing two main groups of strains, amplicons Q01-4, X18-35, and Z04-2 were sequenced, and sequences were deposited in GenBank (Table 3). These sequences were compared, and polymorphic restriction sites were identified. Based on identified sequence differences (single nucleotide polymorphisms and insertions/deletions), restriction enzymes were selected to digest PCR amplicons and develop CAPS markers useful for *T. atroviride* identification (Table 3). In the case of CAPS marker Q01-4 (Q01-4FR amplification product digested with *Bsl*I), a two-band pattern was obtained for 29 strains of *T. atroviride* belonging to group 1, and a single band corresponding to the non-restricted Q01-4 F-4R amplicon was obtained for group 2 strains and TRS18 (Fig. 5). This CAPS marker allows the distinction of strains belonging to *T. atroviride* group 1. For Z04-2 amplification products, *Dra*I digestion resulted in banding patterns reflecting the RAPD-based grouping. However, the products for strain TRS6 and TRS18 were digested and these strains were not classified to the group 1 as in the RAPD study (Fig. 5). We propose that analysis of both CAPS markers can be used to extend ITS/*tef1*-based *T. atroviride* identification and can assist in classification of strains within clade A into one of the two main groups of *T. atroviride*: group 1 (IMI206040-like), characterized by both Q01-4 and Z04-2 amplicons digested, and group 2 (CBS 693.60-like), characterized by both amplicons non-digested. In the case where Q01-4 amplicon is not digested and Z04-2 is digested, additional markers have to be developed to distinguish TRS6-like from TRS18-like strains. Developed CAPS markers have to be further tested on a larger number of strains that will confirm their usefulness in *T. atroviride* genetic studies. The variable RAPD loci found in this study and characterized at the sequence level may be useful in the development of more complex assays, such as multiplex PCR, real-time PCR, or next-generation sequencing (NGS)-based assays, that will be valuable in identification and monitoring of *T. atroviride* strains, as well as in genetic diversity studies including metagenomics approaches and further *T. atroviride* pan-genome understanding.
